# Hypodermic Administration of Carbolic Acid

**Published:** 1882-05

**Authors:** N. B. Kennedy

**Affiliations:** Hillsboro, Texas


### HYPODERMIC ADMINISTRATION OF CARBOLIC ACID,

### FOR THE CURE AND REMOVAL OF FOUL AND ILL-CONDITIONED ULCERS, BOTH INTERNALLY AND EXTERNALLY SITUATED, POISONOUS BITES, HEMORRHOIDS, CARBUNCLES, AND TUMORS.

Read before the Texas Medical Association, at its session, City of Waco, Texas.

BY N. B. KENNEDY, M.D., OF HILLSBORO, TEXAS.

---

Mr. President and Gentlemen:—I wish to offer for your consideration, on this occasion, some of the conclusions I have arrived at, during a period of three years, in the administration, hypodermically, of carbolic acid, for the cure and removal of ulcers, poisonous bites, carbuncles, and tumors.

Carbolic acid, as you all know, is a solid, obtained from the products of the distillation of coal tar, between a temperature of three hundred and four hundred degrees. It was discovered by Runge, in 1834, who gave it the name of carbolic acid; but to Dr. Calvert is due the credit of introducing it into general use in the United States and England.

Carbolic acid may be defined to be an antiseptic anæsthetic, with slight caustic properties, and one of the most powerful disinfectants known. It has a very feeble claim to be recognized as an acid, being more closely allied to alcohol than acids, and, in consequence, is sometimes called phenylic alcohol. Carbolic acid has the power of preventing putrefaction in animal substances, arrests fermentation, and coagulates albumen. Carbolic acid in the liquid form is very slightly irritant; and when applied undiluted to the skin, causes it to turn white, but produces very little pain. In contact with mucous surfaces, it acts in the same manner.

When I commenced, three years ago, experimenting with carbolic acid, hypodermically, it was with a firm belief that I would succeed. The experiments then commenced have ceased to be experiments, but have become established facts. I beg your indulgence while I rehearse to you, in as concise a manner as possible, some of the few out of many successful cases treated hypodermically and locally with carbolic acid.

Case 1.—Mrs. McC., a delicate little woman, I found suffering with hemorrhoids, nearly as large as a goose egg. I injected, hypodermically, five or six drops of Calvert's undiluted carbolic acid into the center of the tumor. The pain was but momentary, the anæsthetic effects of the acid following immediately on withdrawal of the needle. Four more injections effected a complete cure in this case.

Case 2.—I was called, August 19th, 1878, to see J. R. D., one of our most esteemed citizens, suffering with fifteen large carbuncles on his back and neck. He was suffering fearful agony, his family informing me he had not slept five minutes in five days and nights. I at once injected four or five drops of carbolic acid into each of the carbuncles with the happiest effects. In five minutes the old gentleman was in a sound and refreshing sleep, which continued without intermission from 4 o'clock P. M. until 9 o'clock A. M. He awoke from sleep greatly refreshed and without pain; and never had any more pain. Twelve of the carbuncles aborted and three went on to suppuration, or, rather, had commenced to suppurate, when I first saw them. A complete cure followed in three weeks.

CASE 3.—Was called in the evening to see a boy, ten years old, who had been bitten in the morning by a mountain rattlesnake, in the *dorsum* of the foot. On arrival I found the little fellow suffering terribly, with the foot and leg livid and much swollen. I immediately injected five or six drops of the acid, as near into the bite as I could. The relief from pain was so immediate that the little fellow asked me to let him see the "little thing" that stopped his pain so quick. The swelling rapidly disappeared, and he never suffered another moment's pain.

CASE 4.—J. L. F., a well-to-do farmer, consulted me about a tumor on the back of his neck, which had commenced to grow and was paining him. I found a fibro-cellular tumor about the size of a small egg, and injected about twenty drops of undiluted acid into the centre of the tumor, which, in this case suppurated, but in a week or ten days had entirely disappeared, and left no cicatrix behind.

CASE 5.—My friend, Dr. Carmichæl, of Peoria, Hill County, Texas, invited me to see Miss J. A., a young lady, eighteen years old, in the full bloom of womanhood and beauty, for my opinion in reference to a very large tumor in her right breast. Her history was good; she had been in good health all her life. Two years since the *catamenia* was established, synchronous with which she noticed a swelling in the right breast. It had been growing for two years, and was now as large as a child's head. She had never suffered any pain in the gland. I diagnosed it to be a multilocular adenoid tumor. I injected about twenty drops of pure, undilated acid into the most prominent portion of the tumor. The pain was very slight, the anæsthetic effects following immediately the withdrawal of the needle. I used the remedy once a week, from week to week, for two months, at the expiration of which time the tumor had decreased in size from that of a child's head to that of a goose's egg. I now commenced to use the remedy twice a week, and at this writing the tumor is about as large as a hen's egg, and the indications are favorable for its entire eradication. (NOTE.—In a month after this paper was read the tumor had entirely disappeared.)

I also use carbolic acid locally with the happiest effects. I was called to see the Rev. Mr. B's wife, who had been confined to her room and bed for two years, and had had various treatments, both from regular and irregular physicians, with both little benefit. An examination, per vaginam, revealed a fearfully ulcerated os and cervix uteri, with an exceedingly offensive discharge and smell from the genitalia. I applied a fifty per cent. solution of the acid to the ulcers three times per week, and directed for her a good alterative tonic; and in three months she was the very picture of health.

A highly nervous young lady came to my residence to have a felon lanced, and was clamorous for chloroform, as she feared the pain would kill her. I bathed her finger in pure, undiluted acid, and lanced the felon almost without pain, much to her astonishment.

In conclusion, then, allow me to advance the opinion that in carbolic acid we have one of the most powerful remedies for good in the whole pharmacopœia, and one at no distant day destined to supersede almost all other medical and surgical procedure. An antiseptic and anæsthetic disinfectant, *facile princeps*, in surgical cases.

